# Clinical diversity and molecular mechanism of VPS35L-associated Ritscher-Schinzel syndrome

**DOI:** 10.1136/jmg-2022-108602

**Published:** 2022-09-16

**Authors:** Shiomi Otsuji, Yosuke Nishio, Maki Tsujita, Marlene Rio, Céline Huber, Carlos Antón-Plágaro, Seiji Mizuno, Yoshihiko Kawano, Satoko Miyatake, Marleen Simon, Ellen van Binsbergen, Richard H van Jaarsveld, Naomichi Matsumoto, Valerie Cormier-Daire, Peter J.Cullen, Shinji Saitoh, Kohji Kato

**Affiliations:** 1 Department of Pediatrics and Neonatology, Nagoya City University Graduate School of Medical Sciences and Medical School, Nagoya, Japan; 2 Department of Pediatrics, Nagoya University Graduate School of Medicine, Nagoya, Japan; 3 Department of Biochemistry, Nagoya City University Graduate School of Medical Sciences and Medical School, Nagoya, Japan; 4 Université Paris Cité, Génétique clinique, INSERM UMR 1163, Institut Imagine, Hôpital Necker Enfants Malades (AP-HP), Paris, France; 5 School of Biochemistry, Faculty of Life Sciences, University of Bristol, Bristol, UK; 6 Department of Pediatrics, Aichi Developmental Disability Center, Kasugai, Japan; 7 Department of Pediatrics, Toyota Memorial Hospital, Toyota, Japan; 8 Department of Human Genetics, Yokohama City University School of Medicine Graduate School of Medicine, Yokohama, Japan; 9 Department of Clinical Genetics, Yokohama City University Hospital, Yokohama, Japan; 10 Department of Genetics, University Medical Centre Utrecht, Utrecht, The Netherlands; 11 Department of Genetics, Research Institute of Environmental Medicine, Nagoya University, Nagoya, Japan

**Keywords:** genetics, medical

## Abstract

**Purpose:**

The Retriever subunit *VPS35L* is the third responsible gene for Ritscher-Schinzel syndrome (RSS) after *WASHC5* and *CCDC22*. To date, only one pair of siblings have been reported and their condition was significantly more severe than typical RSS. This study aimed to understand the clinical spectrum and underlying molecular mechanism in VPS35L-associated RSS.

**Methods:**

We report three new patients with biallelic *VPS35L* variants. Biochemical and cellular analyses were performed to elucidate disease aetiology.

**Results.:**

In addition to typical features of RSS, we confirmed hypercholesterolaemia, hypogammaglobulinaemia and intestinal lymphangiectasia as novel complications of VPS35L-associated RSS. The latter two complications as well as proteinuria have not been reported in patients with *CCDC22* and *WASHC5* variants. One patient showed a severe phenotype and the other two were milder. Cells established from patients with the milder phenotypes showed relatively higher VPS35L protein expression. Cellular analysis found VPS35L ablation decreased the cell surface level of lipoprotein receptor-related protein 1 and low-density lipoprotein receptor, resulting in reduced low-density lipoprotein cellular uptake.

**Conclusion:**

VPS35L-associated RSS is a distinct clinical entity with diverse phenotype and severity, with a possible molecular mechanism of hypercholesterolaemia. These findings provide new insight into the essential and distinctive role of Retriever in human development.

**WHAT IS ALREADY KNOWN ON THIS TOPIC**

- The Retriever subunit *VPS35L* is the third responsible gene for Ritscher-Schinzel syndrome (RSS) after *WASHC5* and *CCDC22*.
- To date, only one pair of siblings have been reported and their condition was significantly more severe than typical RSS.

**WHAT THIS STUDY ADDS**

- This report expands the clinical spectrum of patients with pathogenic *VPS35L* variants, and identifies both overlapping and distinctive features compared with patients with CCC and WASH complex dysfunction.
- We also demonstrate a possible molecular mechanism for aberrant lipid metabolism observed in these patients.

**HOW THIS STUDY MIGHT AFFECT RESEARCH, PRACTICE OR POLICY**

- This study contributes to the establishment of a disease concept for VPS35L-associated RSS, and provides new insight into the essential and distinctive role of Retriever in human development.

## Introduction

The plasma membrane of human cells contains thousands of integral membrane proteins that play essential roles in a variety of cellular functions including signalling, ion and nutrient transport, cell adhesion and cell polarity.^1^ The localisation of integral cell surface proteins is a highly dynamic process that results from a balance in protein biosynthesis and secretion through the biosynthetic pathway, which is coordinated through sorting and transport of internalised proteins within the endosomal network. For the latter, internalized integral proteins are either sorted for transport to the lysosome for degradation or they avoid this fate and undergo recycling to the cell surface.^2^ It is increasingly clear that perturbation in the balance of these pathways, and their resulting effects on the residency of cell surface proteins, is an underlying feature of human disease.^3^

Retriever is a stable protein complex composed of three subunits, VPS35L, VPS29 and VPS26C. Retriever localises to endosomes from where it regulates the sorting and recycling of internalised integral proteins back to the cell surface through association with accessory proteins that include the cargo adaptor sorting nexin 17 (SNX17), the CCC (CCDC93, CCDC22 and COMMD) complex and the WASH complex, a pentameric protein complex that includes WASHC4 and WASHC5 subunits.^4^ The SNX17-Retriever-CCC-WASH sorting pathway controls the sorting and recycling of numerous cell surface proteins, including integrins, such as integrin β1 (ITGB1), and low-density lipoprotein receptor-related protein 1 (LRP1). As such, depletion of genes in this sorting and recycling pathway leads to a decrease in the expression of these cell surface proteins.^4–7^

In the SNX17-Retriever-CCC-WASH sorting pathway, *WASHC4* has been reported as a causative gene for autosomal recessive intellectual disability (MIM615817), and *CCDC22* and *WASHC5* are known as responsible genes for Ritscher-Schinzel syndrome (RSS, MIM220210 and MIM300963), also known as 3C syndrome due to its triad of clinical features including craniofacial features, cerebellar anomalies and cardiac defects.^8–12^ We previously reported siblings with biallelic loss-of-function variants in *VPS35L*, which is listed as the third responsible gene for RSS (MIM619135). The siblings showed overlapping phenotypes that were similar to 3C syndrome, including the triad of RSS, as well as global developmental delay, skeletal malformation and ophthalmological malformation. That said, they exhibited a more severe disease condition compared with patients with RSS that had *WASHC5* or *CCDC22* pathogenic variants. The elder girl died in the infantile period due to sudden cardiac arrest while the younger boy presented with a variety of severe complications, including short stature (−6 SD), microphthalmia, coloboma, proteinuria, severe global developmental delay and skeletal malformation that included vertebral body hypo-ossification, breast bone aplasia, shortened ulnae with radial bowing, short limbs and chondrodysplasia punctata.

To better understand the clinical manifestations of VPS35L-associated RSS, more clinical information is required. While impairment of cell surface protein recycling and the subsequent reduction of protein expression level is likely to be a molecular cause of the observed clinical phenotypes, the underlying molecular mechanisms in these patients have not been well investigated. In this study, we sought to understand the clinical features of patients with *VPS35L* variants, and to uncover the molecular dysfunction in VPS35L and the Retriever complex that leads to human disorders.

## Materials and methods

### Overview of exome sequencing

Trio-whole exome sequencing was performed by our local genome diagnostic laboratory and processed according to standardised diagnostic pipelines.^13 14^

Two unrelated patients (patient-3 and patient-4) were recruited via GeneMatcher.^15^

### Plasmid construction and cell culture

Human VPS35L cDNA was cloned into pLVX-GFP-puro lentiviral vector. Gibson assembly (NEW ENGLAND BioLabs) was performed to generate VPS35L variant constructs. All constructs were verified by DNA sequencing. To produce lentivirus, the pLVX-VPS35L-GFP vector was co-transfected into HEK293T cells with the Pax2 and pMD2G helper plasmids using polyethylenimine(PEI). Human neuroblastoma H4 cell lines were transduced with lentivirus and cultured with puromycin for positive selection.^16^

### Protein interaction and western blot analysis

For immunoprecipitation of expressed VPS35L-GFP, cells prepared in 15 cm dishes with 100% confluency were lysed in Tris-based immunoprecipitation buffer (50 mM Tris-HCl, pH 7.4, 0.5% NP-40, and Roche protease inhibitor cocktail) before being subjected to GFP trap beads (gta-20, ChromoTek). Lysates were incubated with GFP trap beads for 1 hour at 4°C, then beads were washed three times with immunoprecipitation buffer. Proteins were resolved on NuPAGE 4%–12% precast gels (NP0336BOX, Invitrogen) and then transferred onto polyvinylidene fluoride membranes (10600029, GE Healthcare). After blocking with 5% skim milk in Phosphate buffered saline with Tween-20(PBST), membranes were incubated with primary antibodies, followed by incubation with Alexa Fluor secondary antibodies (680 and 800, Invitrogen). The protein bands were visualised using an Odyssey infrared scanning system (LI-COR Biosciences).^4^

For the immunoprecipitation assay between low-density lipoprotein receptor (LDLR) and VPS35L proteins, 2.5×10^6^ HEK293T cells were plated on 10 cm dishes, and hemagglutinin(HA)-VPS35L and FLAG-LDLR, or FLAG-LDLR were transfected with Lipofectamine 3000 (Thermo Fisher Scientific). The FLAG-LDLR construct was a kind gift from Dr Bart van de Sluis (University of Groningen, The Netherlands). For HA-tag or FLAG-tag immunoprecipitation, lysates were cleared by centrifugation and then incubated with 20 μL of anti-HA-tag pAb-Agarose (561-8; MBL) or anti-DDDDK-tag pAb-Agarose (PM020-8; MBL) for 1 hour at 4°C. The agarose was washed three times with lysis buffer and proteins were eluted with sodium dodecyl sulfate sample buffer.^17^

Primary antibodies used for western blot analysis experiments were as follows: anti-VPS29 (sc-398874; Santa Cruz Biotechnology), anti-VPS35L (ab97889; Abcam), anti-GAPDH (#5174; Cell Signaling Technology), anti-DYKDDDDK-tag (#2368; Cell Signaling Technology), anti-HA-tag (M180-3S; MBL) and anti-VPS26C (ABN87; Merck KGaA).

### Biotinylation assay

To assess the cell surface protein expression of LDLR and LRP1, 3T3 cells of VPS35L-WT, VPS35L-KO or VPS35L-KD were cultured in Dulbecco’s Modified Eagle Medium (DMEM) with 10% lipid protein-deficient human serum (LPDS, #LP4, Merck) for 12 hours, and then the biotinylation assay was performed according to the manufacturer’s guidelines (#K295, BioVision). Briefly, cells were washed and incubated with a solution of Sulfo-NHS-SS-Biotin for 30 min. Cells were then incubated with a quenching buffer for 5 min, then collected by scraping in Tris-Bufferd Saline(TBS) before centrifugation, resuspension in Lysis Buffer, incubation for 30 min, and subsequently centrifuged. A fraction of the supernatant was used as whole cell lysate, and the remainder was incubated with streptavidin beads for 1hr. Beads were collected and resuspended in elution buffer, and used as cell surface protein [17].

### DiI-LDL uptake assay

3T3 cells were plated in 24-well plates, then cultured in DMEM supplemented with 10% LPDS for 16 hours. The medium was changed with DiI-LDL (5 μg/mL) containing 5% LPDS/DMEM, and after 30 min cells were fixed and mounted with 4',6-diamidino-2-phenylindole(DAPI) (D9542-1MG;Merck). Images were acquired with a Nikon A1R. Fluorescence intensity was quantified using ImageJ software (National Institutes of Health) and normalised to the number of DAPI nuclei per image; 30 fields per condition were recorded.^17^

### Characteristic analyses of blood samples

Blood from patients and healthy volunteers was collected in vacutainer blood collection tubes, centrifuged to isolate serum then stored at −80°C until use. Lipoprotein analyses were performed using a HPLC gel-permeable column (Skylight PakLP1-AA gel permeation column, 300 mm×4.6 mm inner diameter) chromatography system and lipid subfractions were analysed using Gaussian approximation curves (LipoSEARCH, Skylight Biotech, Akita, Japan) as previously described.^18 19^ Cholesterylester (CE) was calculated by the difference between the total cholesterol level and the free cholesterol level. The subtracted amounts were multiplied by the average CE/cholesterol molecular weight ratio 1.6.^20^ Apolipoprotein E levels were measured using a turbidimetric immunoassay^21^ by SRL (Tokyo, Japan). Cholesterylester transfer protein (CETP) mass was detected using an ELISA kit (STA-614; Cell Biolabs).

### Additional methods

Immunofluorescence staining, generation of knockout or knockdown cell lines and statistical analysis are described in online supplemental materials and methods.

10.1136/jmg-2022-108602.supp4 Supplementary data

## Results

### Genetic analysis and clinical findings

**Figure 1:**
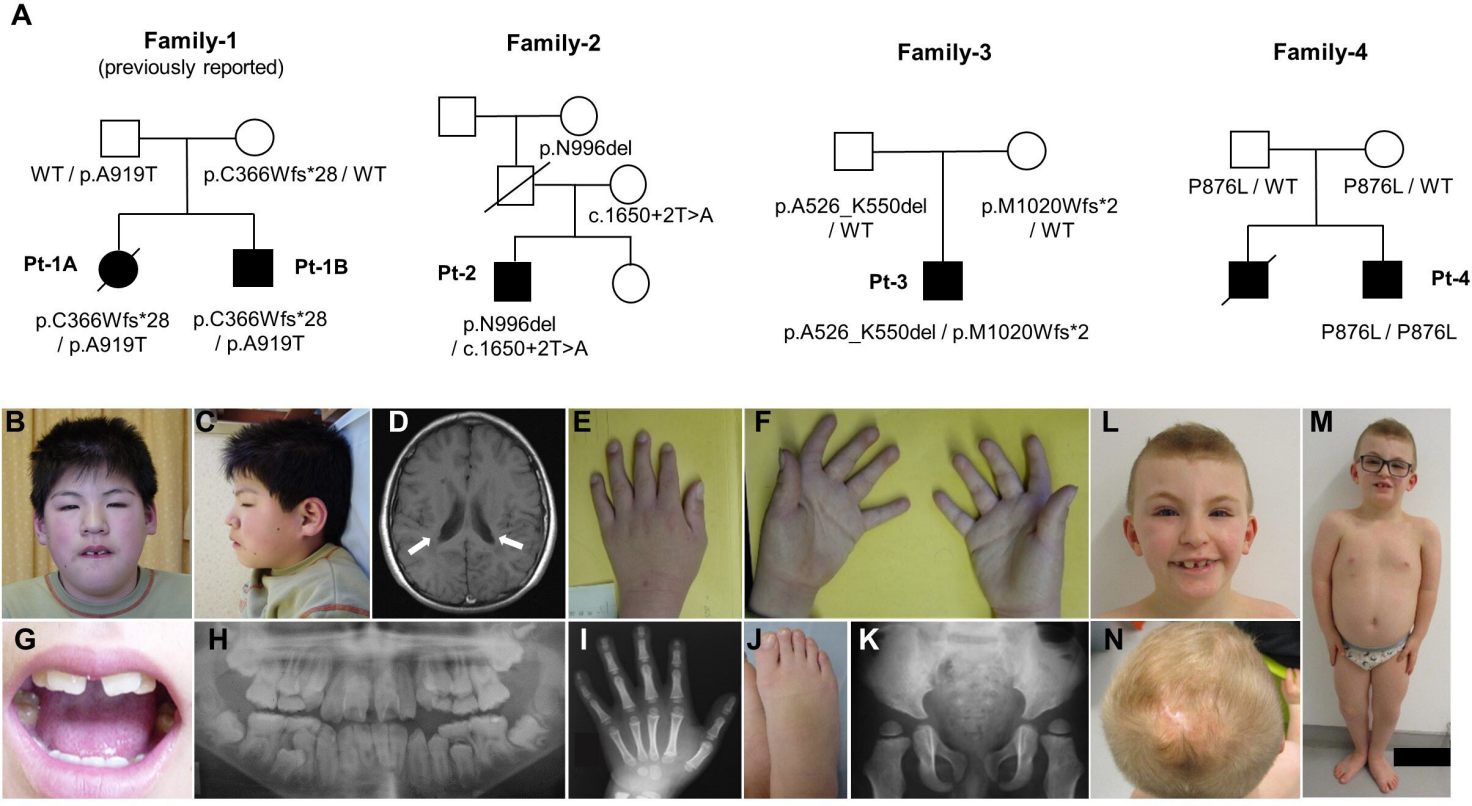
Pedigree analysis and clinical features of patients with VPS35L pathogenic variants. (A) Pedigree analysis of the patients represented by the filled symbol. *VPS35L* genotypes of the family members are given. (B–K) Clinical features of patient (Pt)-2. (B–C) Facial photographs showing bushy and arched eyebrows, broad face, telecanthus, thin upper lips, long philtrum, macrodontia and brachycephaly. (D) Axial-slice of T1-weighted image taken at 15 years of age showing periventricular nodular heterotopia. (E, F, I, J) Short and tapered fingers. X-ray image showing small terminal phalanges, pointed distal ends of proximal and middle phalanges. (G, H) Macrodontia. (K) X-ray image showing hypoplastic ilia, dysplastic acetabulum and small capital epiphysis. (L–N) Clinical features of patient Pt-3. (L–M) Facial photographs showing arched, bushy and wide eyebrows, broad face, thin upper lips, long philtrum and macrodontia. (N) Aplasia cutis congenita on the scalp.

We identified three patients with biallelic *VPS35L* variants through exome sequencing (patient-2, patient-3 and patient-4), all of them are offspring of non-consanguineous healthy parents (figure 1A, online supplemental figure S1A–C). All the identified variants are described in table 1. As for the other candidate, a hemizygous variant (NM_019117.4: c.859A>G, p.Ile287Val) in *KLHL4* was identified in patient-4, but it was considered as uncertain significance. As with the siblings we previously reported (patient-1A, patient-1B), the three new patients showed overlapping clinical features that included global developmental delay, intellectual disability, short stature, brain image findings, craniofacial dysmorphism, ophthalmological malformation, skeletal complications and cryptorchidism (table 1, figure 1B–N). Although no identifiable picture of patient-4 is published here, he presented common craniofacial features that included relative macrocephaly, large anterior fontanelle, hypertelorism and arched eyebrow. It is noteworthy that patients showed diverse severities in their conditions. Among the three patients, patient-4 exhibited the most severe condition with profound intellectual disability, severe short stature (−6 SD) and congenital glaucoma resulting in blindness. His elder brother was affected by the same condition, but a genetic test was not performed, and he died due to sepsis after cardiac surgery for a large ventricular septal defect. Patient-2 and patient-3 showed milder phenotypes with short stature between −2 and −3 SD and mild developmental delay or intellectual disability.

10.1136/jmg-2022-108602.supp1 Supplementary data

**Table 1:** Comparison of clinical features in patients with *VPS35L*, *CCDC22* and *WASHC5* pathogenic variantstable 1

	*VPS35L*					*CCDC22*	*WASHC5*
	Pt-1A (previous case)	Pt-1B (previous case)	Pt-2 (present case)	Pt-3 (present case)	Pt-4 (present case)		
Gender/Age	Female/0 year*	Male/7 years	Male/31 years	Male/8 years	Male/32 years		
Intellectual disability	NA	Severe	Mild	Mild	Profound	Normal to profound	10/10
Postnatal growth retardation	NA	−6.2 SD	−2.1 SD	−2.9 SD	−6.5 SD	−3.7 SD to normal	ND
Brain MRI findings							
Cerebellar dysplasia	+	+	–	–	+	3/8	5/10
Cortical dysplasia	–	+ (PNH)	+ (PNH)	–	+ (Enlarged ventricles)	–	1/10
Calcification	–	–	–	+ (Falx cerebri)	+ (Cerebellum and BG)	–	–
Craniofacial dysmorphism							
Relative macrocephaly	–	+	+	+	+	2/8	10/10
Arched eyebrows	+	+	+	+	+	–	5/10
Thin upper lips	+	+	+	+	–	3/8	5/10
Macrodontia	–	–	+	+	–	ND	ND
Limb abnormalities							
Brachymelia/Brachydactyly	+	+	+	+	–	ND	2/10
Reduced distal creases	–	+	+	+	+	1/8	1/10
Congenital heart diseases	AVSD	–	–	–	VSD	3/8	6/10
Ophthalmological changes							
Glaucoma	+	–	–	+	+	1/8	ND
Coloboma	+ (Choroidal coloboma)	+ (Choroidal coloboma)	–	–	–	ND	3/10
Microphthalmus	–	+ (Optic nerve atrophy)	–	–	–	ND	ND
Proteinuria	NA	+	+	+	NA	ND	ND
Dyslipidaemia	NA	+	+	+	NA	2/8	ND
Increased transaminases	NA	+	+	+	NA	ND	ND
Hypogammaglobulinaemia	NA	+	+	+	NA	ND	ND
Generalised hypotonia	NA	+	+	+	+	3/8	ND
Others	CDP	Epilepsy, CDP, hypoplasia of vertebral body and breast bone	Intestinal lymphangiectasia Hearing impairment	Congenital scalp defect, cutis marmorata	Hearing impairment, ocular albinism, large anterior fontanelle	Hypoplastic lung	Hydrocephaly
Variant 1†	c.2755G>A; p.A919T	c.2755G>A; p.A919T	c.1650+2T>A	c.3057del; p.M1020Wfs*2	c.2627C>T; p.P876L		
Variant 2†	c.1097dup; p.C366Wfs*28	c.1097dup; p.C366Wfs*28	c.2988_2990del; p.N996del	c.1577del; p.A526_K550del‡‡	c.2627C>T; p.P876L		

*Sudden death at infantile period.

†Transcript, NM_020314.5.

‡p.A526_K550del was experimentally confirmed while p.A526Vfs*14 was estimated in silico.

§

–, not present; +, present; AVSD, atrioventricular defect; BG, basal ganglia; CDP, chondrodysplasia punctata; NA, information not available; ND, information not described; PNH, periventricular nodular heterotopia; Pt, patient; VSD, ventricular septal defect.

In addition to the complications reported in the previously reported siblings, we identified some novel complications in VPS35L-associated RSS. All three patients had immunological issues with low serum IgG levels, as was also identified in patient-1B. Patient-4 was diagnosed with common variable immunodeficiency (CVID) as he presented with a recurrent respiratory tract infection along with a low immunoglobulin level. Patient-2 was affected by cryptococcal meningitis at 15 years of age, and diagnosed as hypogammaglobulinaemia with serum IgG below 200 mg/dL. The patient is now receiving regular subcutaneous immune globulin therapy, and he has not developed severe infectious disease since its initiation. In addition, ^99m^Tc-human serum albumin scintigraphy and α1-antitrypsin clearance in patient-2 identified a leakage of proteins from serum to the gastrointestinal tract. Protein leakage was also observed in the urinary system. A urine test strip showed sustained proteinuria in patient-1B, patient-2 and patient-3. Furthermore, serum biochemistry examinations revealed elevated LDL levels in patient-1B, patient-2 and patient-3. These results uncovered a previously unknown clinical phenotype and diversity in patients with VPS35L-associated RSS.

### Protein and mRNA analysis using patient-derived cells

**Figure 2:**
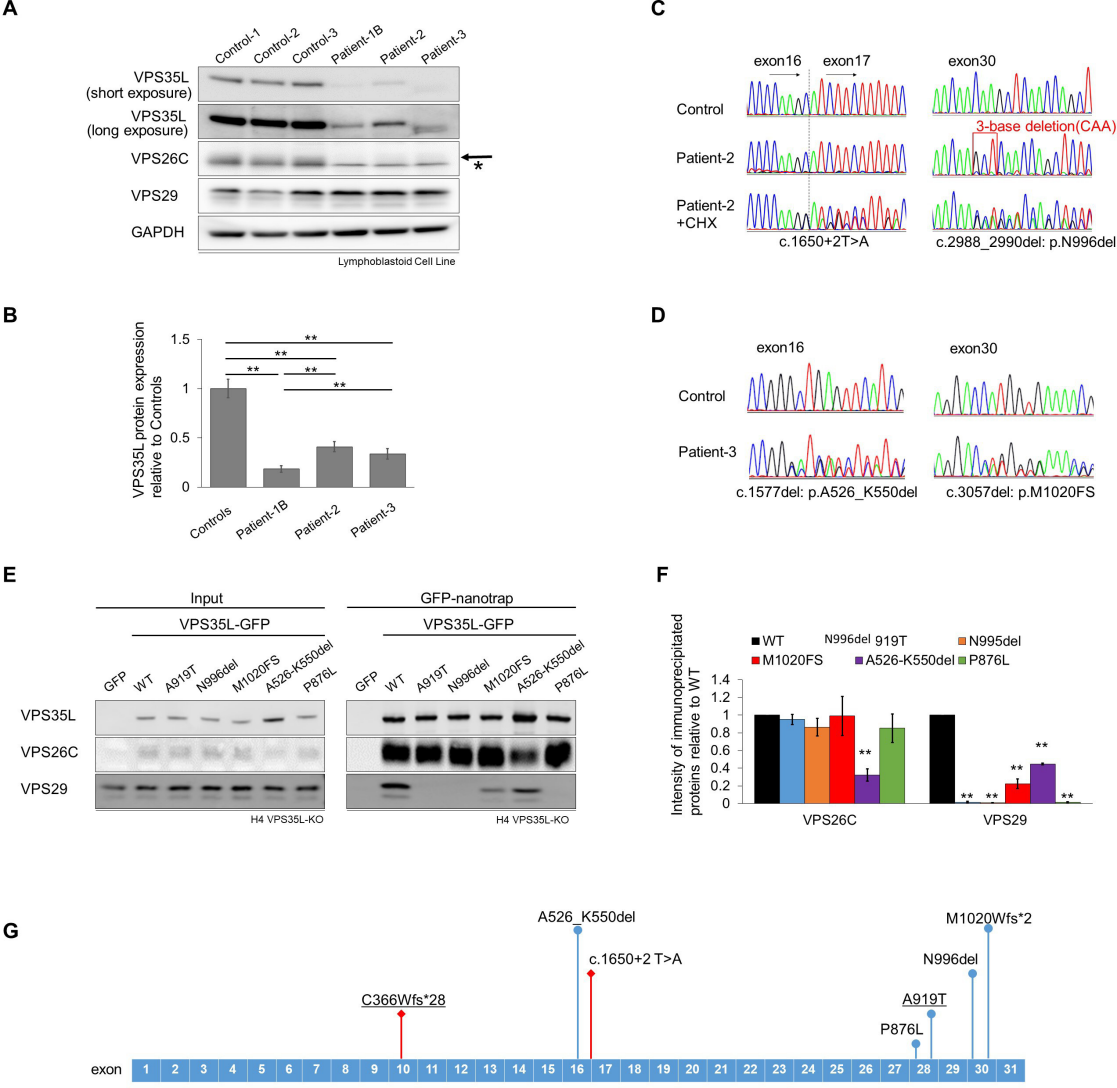
Functional analysis of lymphoblastoid cell lines (LCLs) derived from patients. (A) Representative immunoblots of VPS35L, VPS26C and VPS29 expression in LCLs derived from patients and healthy controls. GAPDH: loading control, arrow: VPS26C, asterisk: non-specific band. (B) Quantification of VPS35L intensity from three independent experiments. (C) cDNA sequence indicated mRNA decay in the splice site variant allele, which was recovered by administration of cycloheximide (CHX). (D) cDNA sequence indicated comparable expression level of each allele with c.1577del or c.3057del. (E) Representative blot of GFP-nanotrap analysis. Wild-type (WT) or identified variants of VPS35L-GFP fusion protein was expressed in VPS35L-KO H4 cell line. (F) Quantification from three independent experiments. Relative density of the bands in immunoprecipitated proteins was determined relative to one at VPS35L-WT. (G) Schematic illustration of VPS35L exon structure together with identified variants. Variants with a significantly reduced mRNA level are shown with red and square-tipped lines, and variants with normal mRNA expression are presented with blue and circle-tipped lines. The c.1577del variant was predicted to be translated to p.A526Vfs*14 based on in silico analysis, however sequence analysis of cDNA identified alternative splicing that resulted in a 75 bp in-frame deletion, which was predicted to be translated to p.A526_K550del. Bar graphs (B) and (H), means and SEM are shown, **p<0.01.

Our previous report indicated that the protein expression levels of VPS35L and VPS26C were considerably decreased in the cells established from patient-1B. As such, we checked the expression levels of protein components of the Retriever complex in EB virus-transformed lymphoblastoid cell lines (LCLs) established from patient-2 and patient-3. This found the expression level of both VPS35L and VPS26C were significantly decreased in all patient cells compared with controls (figure 2A). In addition, it is worth noting that the expression level of VPS35L was significantly lower in patient-1B, who showed a more severe disease form compared with patient-2 and patient-3, who both showed a milder phenotype (figure 2B). These results confirm that disrupted VPS35L protein function is the cause of pathogenicity in this disease condition, while residual VPS35L protein expression level can affect clinical severity.

We next analysed mRNA to understand which allele is translated to protein and is involved in nonsense-mediated mRNA decay (NMD). RT-PCR and Sanger sequence analysis using cDNA obtained from LCLs established from patient-2 showed only the transcript of the 3-base (CAA) deletion (c.2988_2990del), but on the other hand, when cells were treated with cycloheximide (CHX) to prevent NMD, additional sequence appeared from the beginning of exon 17 (figure 2C). TA cloning and sequence analysis found the appearing sequence in CHX-treated cells was the same 41 bases at the beginning of intron 16. These results confirmed that the splice site variant caused an exon extension resulting in NMD (online supplemental figure S1D). Interestingly, mRNA levels of *VPS35L* were not significantly decreased in patient-3-derived cells even though the patient carried two frameshift variants (online supplemental figure S1E). The c.1577del variant generated an alternative splicing product with a 75 bp in-frame deletion, which was thought to be translated into a protein with a 25 amino acid deletion of VPS35L-A526_K550del (figure 2D), and the other c.3057del variant was located just before the last exon, which enabled both alleles to escape from mRNA decay.

### Functional analysis of identified variant proteins

To confirm the pathogenicity of the P876L variant identified in patient-4, and to understand why the protein expression level of VPS35L was significantly decreased in cells established from patient-2 and patient-3, we generated lentivirus expressing VPS35L-GFP fusion proteins of WT and all the identified variants considered to be translated to protein in this cohort, which included N996del (patient-2), M1020FS and A526_K550del (patient-3) and P876L (patient-4), along with A919T (patient-1) as a pathogenic control.

We first checked the localisation of VPS35L wild-type (WT and identified variant proteins. As shown in our previous report,^4^ VPS35L-WT-GFP localised to endosomes as defined by colocalisation with the early endosomal marker EEA1, and all five variant proteins retained the ability to localise to early endosomes (online supplemental figure S2A and S2B).

10.1136/jmg-2022-108602.supp2 Supplementary data

We next checked the capacity of all variant proteins to assemble into the Retriever complex, as we previously reported that the A919T variant lacked binding to VPS29, resulting in protein instability. The two novel variant proteins, N996del and P876L, showed results similar to that observed in A919T, with disrupted binding to VPS29 while binding to VPS26C was not significantly affected (figure 2E, F). The M1020FS variant also showed a significant reduction in VPS29 binding capacity. Interestingly, all four variants that affect binding to VPS29, are located in the C-terminal region of VPS35L (figure 2G). Conversely, only one variant, A526_K550del, which is located in the middle of VPS35L, showed significantly decreased binding to both VPS26C and VPS29. Together. these data suggest that disruption of protein assembly in the Retriever complex is likely to be the cause of pathogenicity, leading to decreased protein stability and a resultant reduction in the efficiency of endosomal integral protein sorting in cells established from these patients.

### Lipoprotein cholesterol profiles and apolipoprotein E, CETP levels in patient serum

**Figure 3:**
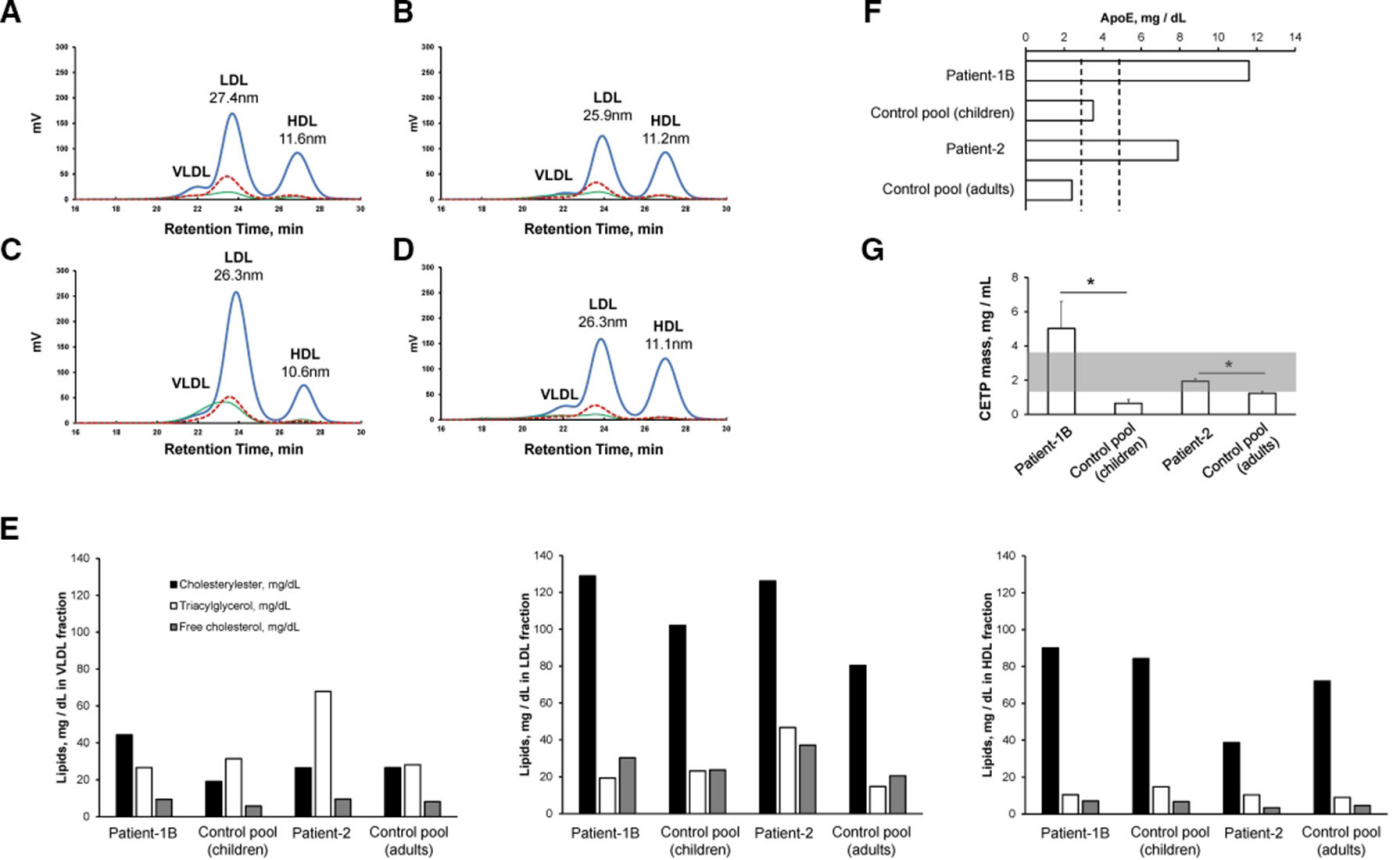
Typical lipoprotein profiles and lipid contents of patients and healthy volunteers. Plasma lipoprotein profiles were analysed by gel permeable HPLC using a tandem SkylightPakLP1-AA gel permeation column equipped with online enzymatic colouring reagent system, LipoSEARCH. (A) Patient-1B, (B) age-matched control pool, (C) patient-2, (D) adult control pool. Bold blue lines indicate cholesterylester content, thin green lines indicate triacylglycerol level, dotted red lines indicate free cholesterol content. The low-density lipoprotein (LDL) and high-density lipoprotein (HDL) diameters at the peak points are indicated. (E) Lipid contents of lipoprotein subfractions of each participant. Solid bars, open bars and grey bars indicate cholesterylester, triacylglycerol and free cholesterol level, respectively. (F) Serum apolipoprotein E (apoE) level are described. Dotted lines indicate threshold amounts of standard serum apoE level in Japanese male populations (2.7–4.3 mg/dL). (G) Serum cholesterylester transfer protein (CETP) mass assayed by an ELISA kit. Standard CETP level is indicated by the coloured zone. Values reported are the mean±SD (n=3). Bar graphs, means and SD are shown; *p<0.05. Statistical analysis was performed by two-way analysis of variance versus control subject.

As three of the patients showed hypercholesterolaemia, lipoprotein lipid contents were examined based on size exclusion HPLC. Total serum CE was substantially increased in patient-1B (264 vs 205 mg/dL in control pool), and was moderately increased in patient-2 (191 vs 181 mg/dL). Figure 3A–D show a representative chromatogram of lipoprotein subclasses. The calculated lipids values are shown in figure 3E. It is noteworthy that very low-density lipoprotein (VLDL)-CE and LDL-CE were higher in patient-1B compared with the age-matched control pool (figure 3A, B). Furthermore, the top LDL particle diameter size was 1.5 nm larger than the control pool serum, which indicates intermediate-density lipoprotein (IDL (small VLDL and large LDL subfractions) are relatively abundant in this patient’s serum. On the other hand, high-density lipoprotein (HDL)-CE levels were comparable with the control pool serum. In patient-2, VLDL-CE was not influenced while LDL-CE was 57% higher than control pool serum (figure 3C, D). Notably, HDL-CE dropped to 38.6 mg/dL in patient-2, which was accompanied by 2.4 times more VLDL-TG and 3.1 times more LDL-TG compared with the control pool. Increased VLDL-CE was often accompanied by increased HDL-CE levels. It is thought that when HDL generation by ABCA1 transporters at hepatocytes is compromised, VLDL secretion is rapidly reversed due to elevated triacylglycerol biogeneration in the liver.^22–26^

To get a detailed understanding of cholesterol homeostasis within the circulation, free cholesterol levels were also examined. In apoB-containing lipoproteins (VLDL and LDL), the free cholesterol levels ratio to CE were relatively constant at 29%±4% and 25%±2%. On the other hand, the free cholesterol ratio decreased to 7%±1% in HDL subclasses due to the higher LCAT activity.^20 27 28^ However, the ratio between patients and the healthy control pool were not significantly different, which indicates the esterification and hydration of cholesterol equilibration in serum was intact in these patients.

To evaluate if the increased lDL (small VLDL and large LDL subfractions) in patient-1B and patient-2 were due to a delay in the apoE-containing lipoprotein catabolic rate, we detected apoE levels in the serum (figure 3F). Patient-1B and patient-2 had 3.3 and 3.2 times more apoE than the control pool, respectively. CETP is another factor that regulates lipoprotein levels within circulation. CETP expression in the liver is regulated by nuclear receptor LXRa,^29^ which orchestrates lipoprotein CE and triacylglycerol equilibration in the serum. In these patients, CETP mass was significantly elevated (figure 3G). In patient-1B, CETP levels were 1.4 times higher than the highest average CETP level. The function of CETP is to recycle CE from the catabolic system by reverse cholesterol transport. Declined LDL and IDL uptake compensates upregulated CETP to increase LDL-CE in these patients.

### VPS35L is essential for lipid metabolism

**Figure 4:**
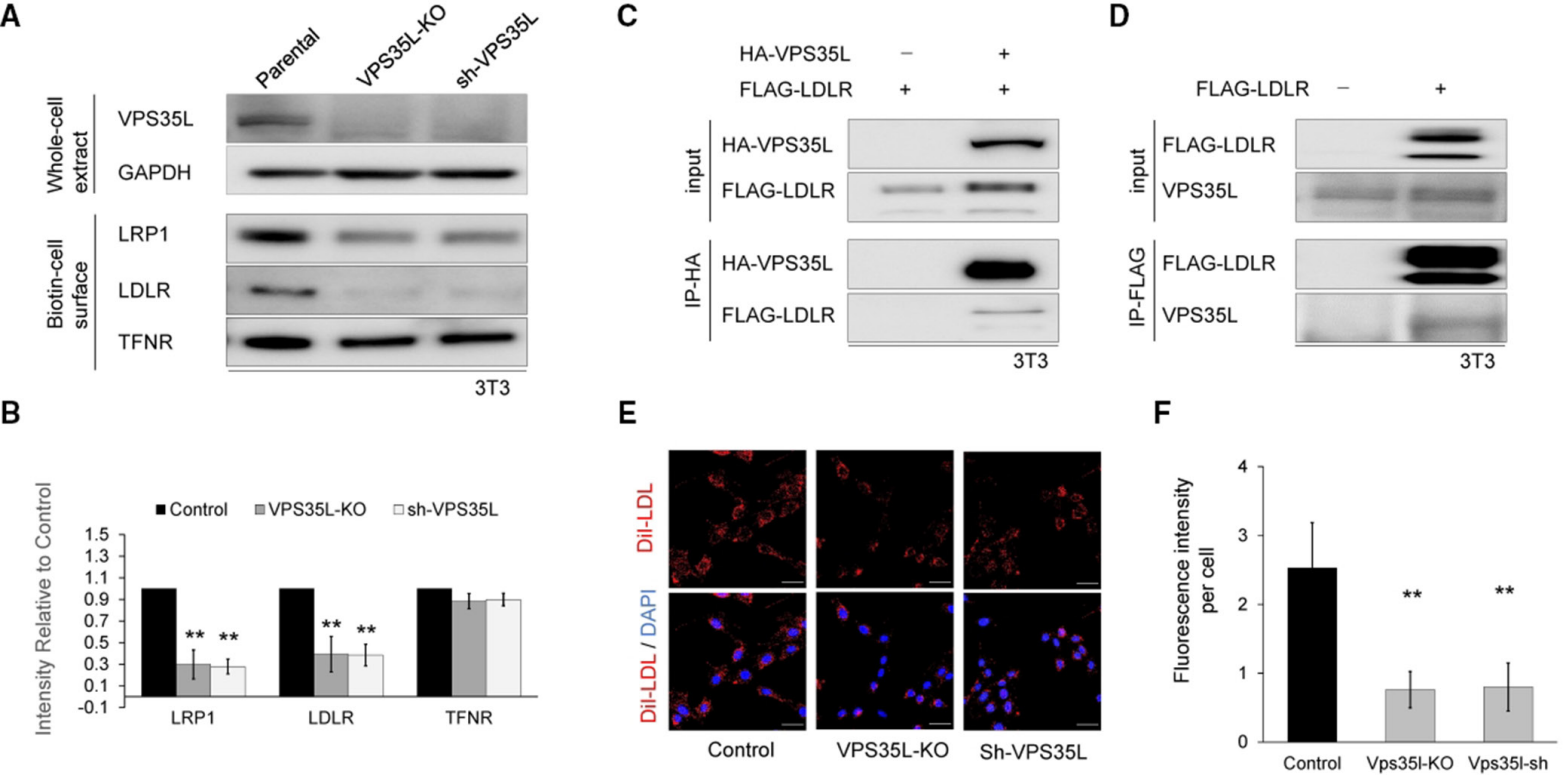
VPS35L deficiency impairs cell surface low-density lipoprotein receptor (LDLR) level and low-density lipoprotein (LDL) uptake. (A) Representative immunoblots of 3T3 parental, VPS35L-knockout (KO) and sh-VPS35L-knockdown cells. glyceraldehyde-3-phosphate dehydrogenase(GAPDH) and transferrin receptor(TFNR) immunoreactivity indicate equivalent loading. (B) Protein intensity of LRP1, LDLR and TFNR relative to parental cells averaged over three independent experiments. (C) HEK293T cells were transfected with either HA-VPS35L alone or HA-VPS35L and FLAG-LDLR. Interaction between HA-VPS35L and FLAG-LDLR was detected via immunoprecipitation with anti-HA antibody. (D) HEK293T cells were transfected with FLAG-LDLR, and interaction with endogenous VPS35L was detected by immunoprecipitation with anti-FLAG antibody. (E) Representative images of DiI-LDL uptake in parental, VPS35L-KO and sh-VPS35L 3T3 cells. Cells were incubated with DiI-LDL for 30 min, followed by DAPI staining and imaging with a fluorescence microscope. (F) DiI intensity was quantified using ImageJ software. We acquired 30 fields percondition, and DiI intensity of each field was normalised to the number of DAPI nuclei. Bar graphs (B) and (F), means and SEM are shown, **p<0.01.

Previous reports found the CCC and WASH complexes act together to facilitate endosomal trafficking of LDLR.^17 30 31^ Given the circulating LDL cholesterol level of patients with VPS35L dysfunction were above the 95th percentile, and the Retriever complex interacts with the CCC and WASH complexes in the endosomal recycling network, we hypothesised that VPS35L may play an essential role in maintaining proper expression levels of LDLR at the cell surface. To test this possibility, we generated VPS35L-knockout and VPS35L-knockdown cell lines in 3T3, using CRISPR/Cas9 and shRNA, respectively. Western blot analysis confirmed that both VPS35L-knockout and VPS35L-knockdown cell lines showed efficient depletion of VPS35L (figure 4A). Also, both VPS35L depleted cells showed decreased cell surface level of LRP1, an integral membrane protein that is sorted through the endosomal network by the SNX17-Retriever-CCC-WASH pathway (figure 4A, B). Moreover, the cell surface level of LDLR was also markedly decreased in both VPS35L depleted cells. These results are consistent with Retriever facilitating the endosomal sorting of the LDLR. Next, we investigated whether VPS35L interacts with LDLR. In immunoprecipitation and immunoblot assays, FLAG-LDLR was immunoprecipitated with HA-VPS35L, and endogenous VPS35L was also immunoprecipitated with FLAG-LDLR (figure 4C, D). To investigate whether the reduction of cell surface levels of LRP1 and LDLR in VPS35L depleted cells affected LDL uptake, we added DiI-LDL to the media. VPS35L depletion markedly diminished DiI-LDL cellular uptake (figure 4E, F). Together, these results indicated that VPS35L play an essential role in maintaining cell surface levels of LRP1 and LDLR most likely via endosomal sorting, and that dysfunction of VPS35L leads to impaired LDL uptake due to decreased cell surface levels of lipoprotein receptor proteins LDLR and LRP1.

## Discussion

Prior to this study, only one pair of siblings with VPS35L pathogenic variants had been described, who presented with a severe form of congenital malformation syndrome. This study has identified VPS35L-associated RSS as a clinical entity, which presents with diverse phenotype and severity. Our cellular analysis suggests that depletion of VPS35L, and loss of the functional Retriever endosomal sorting complex, leads to a decreased cell surface expression of LDLR and LRP1, resulting in impairment of normal LDL uptake. This likely describes the underlying molecular mechanism of hypercholesterolaemia present in these cases of VPS35L-associated RSS.

A review of clinical manifestations in this case series and previously reported patients with either *VPS35L*, *CCDC22* or *WASHC5* pathogenic variants is presented in table 1 and online supplemental figure S3.^9–12 17^ The previously reported siblings with *VPS35L* variants comprised an elder girl who died in the infantile period due to sudden cardiac arrest, and a younger boy who presented a variety of severe complications.^5^ While their clinical manifestation appeared to be more severe than typical phenotypes reported in 3C/RSS cases, a greater number of cases is required to build a more complete understanding of patients with VPS35L-related disorders. In this article, we described three patients with VPS35L pathogenic variants. Their clinical manifestations suggested overlapping clinical features with patients with Retriever, CCC and WASH complex dysfunction. To our knowledge however, the primary intestinal lymphangiectasia and hypogammaglobulinaemia observed in this case series have not previously been reported in patients with pathogenic variants in *CCDC22* or *WASHC5*. It is worth noting that proteinuria was identified in three patients with VPS35L pathogenic variants, which has not been reported in patients with 3C/RSS. These results provide a better clinical understanding of patients with VPS35L pathogenic variants. To determine whether the clinical manifestation between patients with Retriever, CCC or WASH complex dysfunction are distinct will require more cases to generate insight into the distinctive functional roles between these complexes.

10.1136/jmg-2022-108602.supp3 Supplementary data

In this case series, patient-2 and patient-3 exhibited a milder phenotype compared with patient-1B and patient-4. Our functional analysis found cells established from patient-1B expressed significantly lower protein levels of VPS35L compared with cells isolated from patient-2 and patient-3. This is consistent with VPS35L protein levels affecting clinical severity. GFP-nanotrap analysis showed that A919T, N996del and P876L variant proteins were very similar functionally in terms of their lack of binding to VPS29. Yet cells derived from patient-2 with the N996del variant showed higher expression of VPS35L compared with patient-1B. Further study is required to fully understand why patient-2-derived cells express higher protein levels of VPS35L even through the mRNA and GFP-nanotrap analyses showed very similar results.

Previous reports suggest the CCC and WASH complexes facilitate trafficking of lipoprotein receptor proteins including LRP1 and LDLR.^17 30 31^ However, it has not been determined if the Retriever complex is also required for recycling of these proteins. Here, analysis of patient serum found dysregulated lipid homeostasis, and subsequent cellular analysis confirmed that Retriever is also required to facilitate trafficking of LRP1 and LDLR. This suggests that cell surface protein recycling regulated by Retriever is essential for normal cell function and human development, and there is a yet unknown association between ablated cell surface proteins and clinical phenotype in patients with VPS35L dysfunction.

In conclusion, this report expands the clinical spectrum of patients with pathogenic VPS35L variants, and identifies both overlapping and distinctive features compared with patients with CCC and WASH complex dysfunction. We also demonstrate a possible molecular mechanism for aberrant lipid metabolism observed in these patients. These findings provide new insight into the essential and distinctive role of Retriever in human development.

## Data Availability

All data relevant to the study are included in the article or uploaded as supplementary information.
