# Supplementary material for: Clinical diversity and molecular mechanism of VPS35L-associated Ritscher-Schinzel syndrome
Source: J Med Genet. 2022 Sep 16;60(4):359–67. doi: 10.1136/jmg-2022-108602 (PMC10086474; doi:10.1136/jmg-2022-108602)
Supplement: Supplementary data [file jmg-2022-108602supp004.pdf]

SUPPLEMENTARY MATERIALS AND METHODS

Immunofluorescence staining

Cells were fixed in 4% paraformaldehyde in PBS for 20 min at room temperature followed by permeabilization in 0.1% Triton X-100 for 5 min. After blocking in 3% BSA (Cat.# 05482, Sigma), cells were incubated in anti-EEA1 (Cat.# 610456, BD) primary antibody for 1 hr at room temperature, followed by incubation for 30 min at room temperature in secondary antibodies (Alexa Fluor® 568, Invitrogen).

Generation of knock-out (KO) or knock-down (KD) cell lines

3T3 and H4 cell lines lacking VPS35L were generated using CRISPR/Cas9 technology. The target sequence was TTTCAAGATCGCTTCCATCCGGG in exon 10 for 3T3, and CAGCGTCTTACCAGGTAATGCGG in exon9-intron9 for H4. Clones were isolated and gene disruption was validated by PCR-based sequencing and western blot. A 3T3 cell line of VPS35L knock-down was generated using VPS35L-shRNA-expressing lentivirus (Merck). 3T3 cells were incubated with virus-containing medium for 24 hr, then cells were cultured in puromycin containing medium for more than two weeks to select for shRNA expressing cells.

Statistical analysis

Results are presented as the mean ± standard error measurement (s.e.m). A two-sided Student's

Otsuji S, *et al. J Med Genet* 2023; 60:359–367. doi: 10.1136/jmg-2022-108602

t-test was performed to compare the means between two groups. When the means of three groups were to be compared, one-way analysis of variance (ANOVA) with post hoc Tukey's or Dunnet's honestly significant difference calculator test was used. Statistics were calculated using EZR (Saitama Medical Center, Jichi Medical University). $P < 0.05$ was considered significant.

Otsuji S, *et al. J Med Genet* 2023; 60:359–367. doi: 10.1136/jmg-2022-108602
