# Supplementary material for: Clinical diversity and molecular mechanism of VPS35L-associated Ritscher-Schinzel syndrome
Source: J Med Genet. 2022 Sep 16;60(4):359–67. doi: 10.1136/jmg-2022-108602 (PMC10086474; doi:10.1136/jmg-2022-108602)
Supplement: Supplementary data [file jmg-2022-108602supp001.pdf]

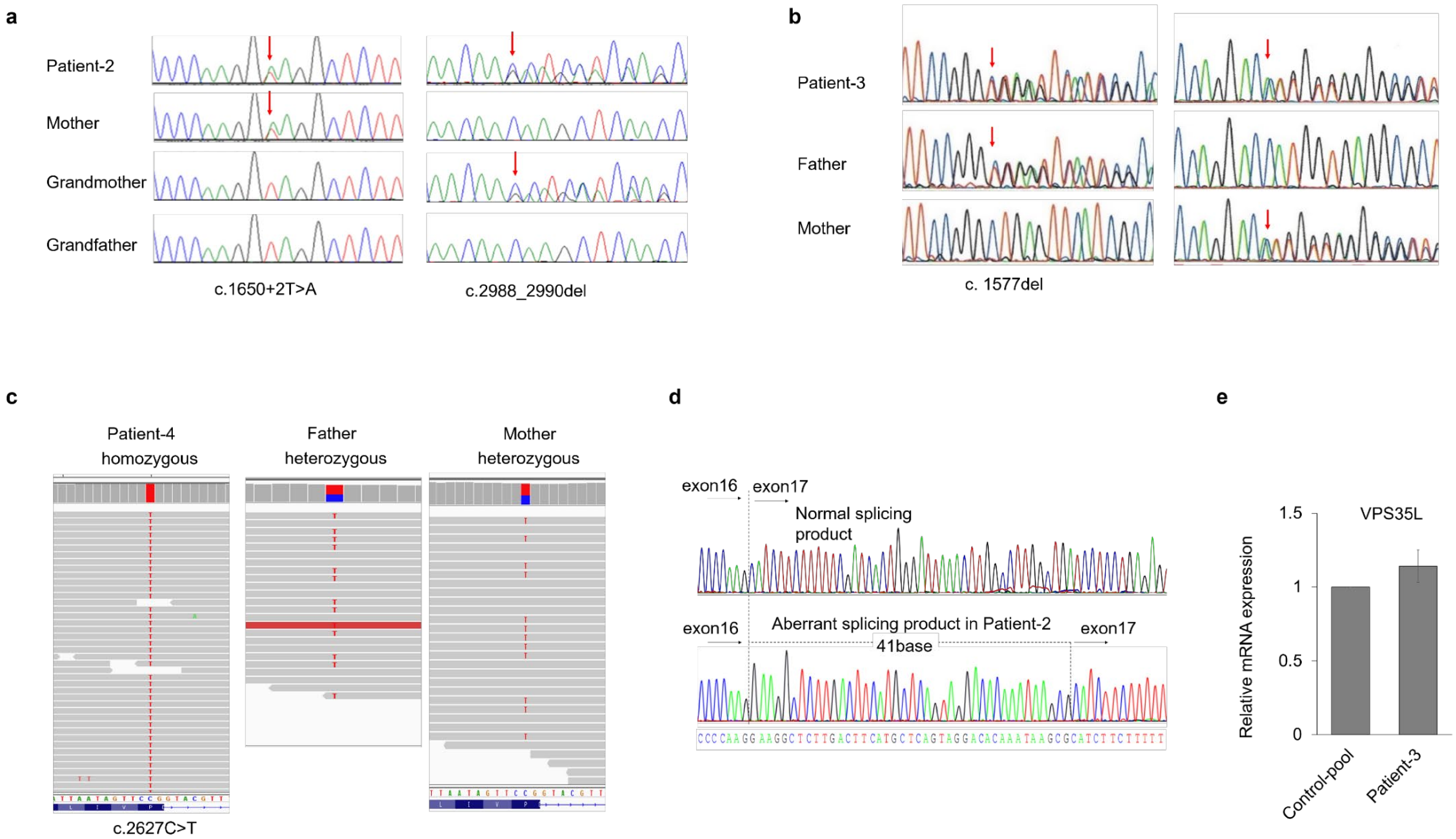

Figure S1. Sequence analysis of DNA and cDNA extracted from cells derived from patients.

(a-c) Genomic DNA sequence chromatograms or snapshot of IGV illustrate the biallelic variants in VPS35L: c.1650+2T>A, c.2988_2990del in patient-2; c.1577del, c.3057del in patient-3 and c.2627C>T in patient-4 (NM_020314.5). (d) TA cloning and sequence analysis of cDNA reverse transcribed from RNA extracted from cells derived from patient-2, which was treated with cycloheximide to block mRNA decay. (e) mRNA Expression level of VPS35L in lymphoblastoid cell line established from either healthy control or patient-3. Bar graphs, means and s.e.m are shown.
