# Supplementary material for: Clinical diversity and molecular mechanism of VPS35L-associated Ritscher-Schinzel syndrome
Source: J Med Genet. 2022 Sep 16;60(4):359–67. doi: 10.1136/jmg-2022-108602 (PMC10086474; doi:10.1136/jmg-2022-108602)
Supplement: Supplementary data [file jmg-2022-108602supp002.pdf]

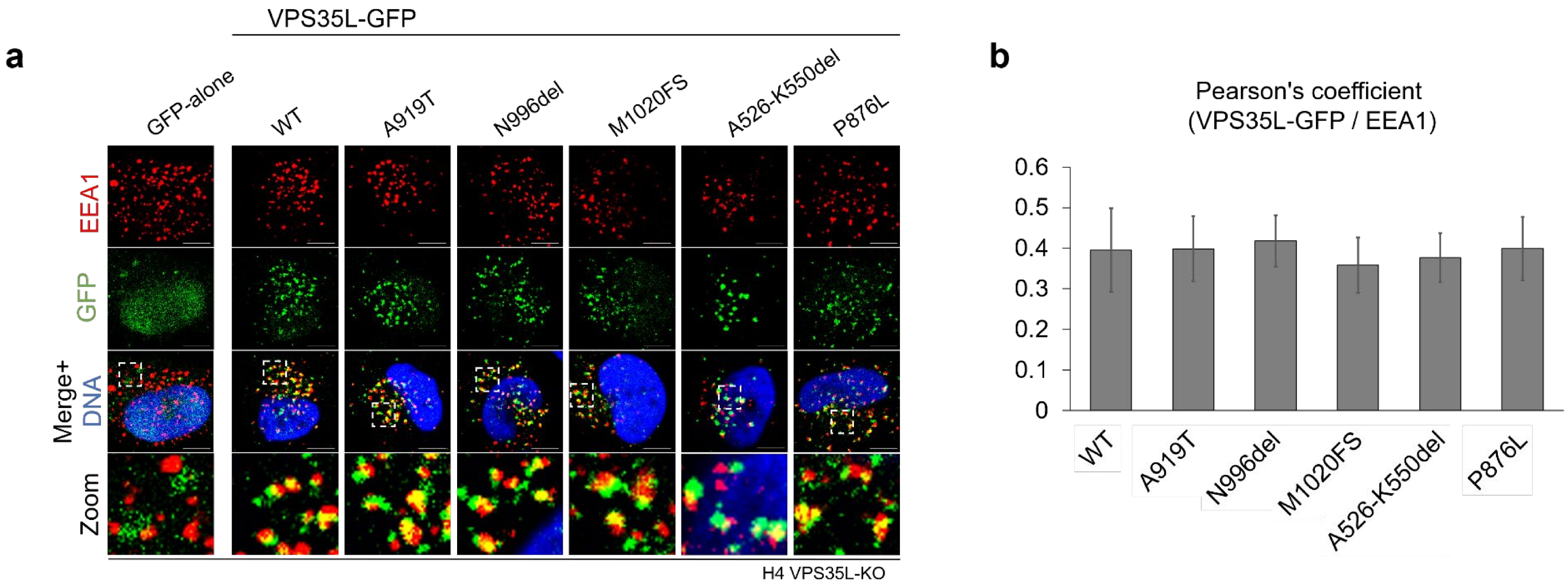

Figure S2. Colocalization analysis of VPS35L-wildtype and mutant proteins.

(a) Immunofluorescence staining of endogenous early endosomal marker EEA1 (red) in H4 glioma cell lines transfected with GFP or VPS35L-GFP expressing lentivirus. Scale Bars, 10μm. (b) Quantification of colocalization of EEA1 and VPS35L-GFP from three independent experiments (n = 30 cells analyzed). Bar graph, means and s.e.m are shown.
