# Supplementary material for: Clinical diversity and molecular mechanism of VPS35L-associated Ritscher-Schinzel syndrome
Source: J Med Genet. 2022 Sep 16;60(4):359–67. doi: 10.1136/jmg-2022-108602 (PMC10086474; doi:10.1136/jmg-2022-108602)
Supplement: Supplementary data [file jmg-2022-108602supp003.pdf]

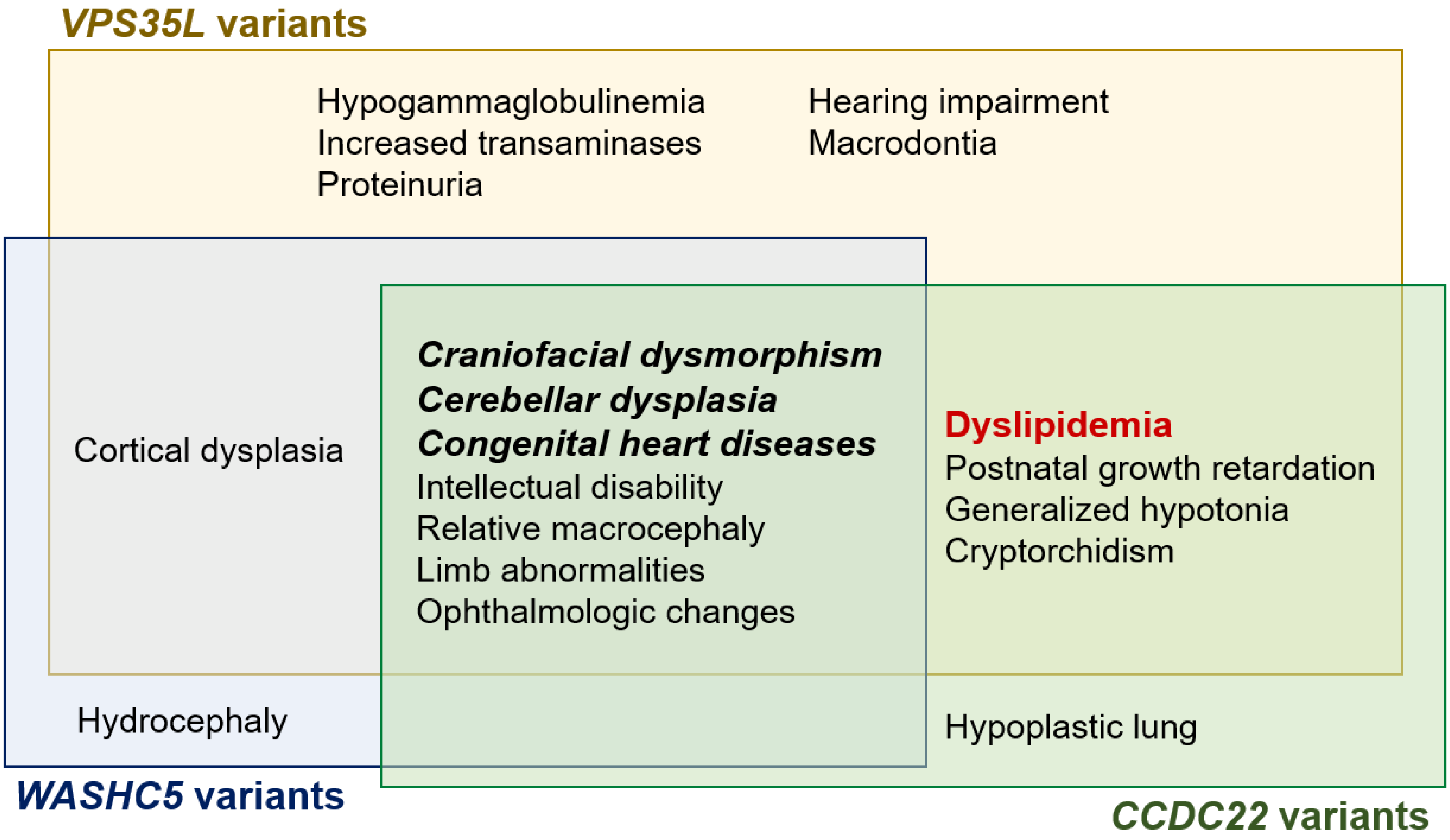

Figure S3. Venn diagram to show clinical overlap and diversity in VPS35L-, WASHC5-, and CCDC22-associated Ritscher-Schinzel syndrome (RSS). Clinical manifestations reported in VPS35L-, WASHC5-, or CCDC22-associated RSS are summarized in this Venn diagram. The bold and italic text represent classical triad of RSS, also known as 3C (cranio-cerebello-cardiac) syndrome. Dyslipidemia is highlighted in bold red text because its pathomechanism was intensively studied in this report.
